# Relation between anion gap and 28-day all-cause mortality in patients with chronic total coronary occlusion: A retrospective cohort analysis

**DOI:** 10.1097/MD.0000000000050537

**Published:** 2026-09-04

**Authors:** Jun Yang, Min Huang, Guofeng Zhu, Zhonglan Cai, Guang Tu

**Affiliations:** a Department of Cardiovascular Medicine, Nanchang County People’s Hospital, Nanchang, Jiangxi, China; b Department of Cardiovascular Medicine, The Second Affiliated Hospital of Nanchang University, Nanchang, Jiangxi, China; c Department of Respiratory and Critical Care Medicine, The Second Affiliated Hospital of Nanchang University, Nanchang, Jiangxi, China; d Department of Cardiology, Lichuan People’s Hospital, Fuzhou, Jiangxi, China.

**Keywords:** anion gap, chronic total coronary occlusion, intensive care unit, MIMIC-IV database, mortality

## Abstract

Chronic total coronary occlusion (CTO) is a critical condition associated with poor outcomes in patients with coronary artery disease. Anion gap, a readily available laboratory marker, has been linked to various clinical outcomes, but its relationship with mortality in CTO patients is not well established. This study aimed to investigate the association between anion gap and 28-day all-cause mortality in patients with CTO admitted to the intensive care unit (ICU). We conducted a retrospective cohort study using data from the medical information mart for intensive care, version IV database (version 3.1), spanning from 2008 to 2019. Patients with CTO admitted to the ICU were included, and those with missing anion gap data or non-first-time ICU admissions were excluded. We used univariate and multivariate Cox regression models to evaluate the impact of anion gap on 28-day all-cause mortality. Adjustments were made for various clinical and laboratory parameters. A total of 830 patients were included in the analysis. The mean age was 69.4 years, and 76.5% were male. Anion gap was significantly associated with 28-day all-cause mortality in both univariate (hazard ratio [HR] 1.09, 95% confidence interval [CI] 1.05–1.13, *P* < .001) and multivariate analyses (HR 1.15, 95% CI 1.10–1.20, *P* < .001). The risk increased across quartiles, with the highest risk observed in the highest quartile (Q4, HR 21.22, 95% CI 2.78–161.93, *P* = .003). Subgroup analysis revealed significant interactions with gender and heart failure. Anion gap is a significant predictor of 28-day all-cause mortality in patients with CTO admitted to the ICU. Our findings suggest that monitoring anion gap could be clinically useful for risk stratification in this patient population. Further studies are needed to confirm these results and explore potential mechanisms.

## 1. Introduction

Chronic total coronary occlusion (CTO), characterized by a complete and persistent blockage of a coronary artery, is a challenging condition that often leads to severe ischemia and adverse cardiac outcomes, including heart failure and sudden cardiac death.^[1,2]^ Despite advancements in interventional cardiology, patients with CTO continue to face a high risk of mortality, particularly when admitted to the intensive care unit (ICU).^[3,4]^ Early identification of patients at higher risk of mortality is crucial for tailored management and improved outcomes.

Anion gap, calculated as the difference between the sum of anions and cations in the blood, is an easily obtainable laboratory parameter that has been associated with various clinical conditions, including sepsis, renal dysfunction, and metabolic disturbances.^[5–9]^ In critical illness, an elevated anion gap can reflect underlying pathophysiological processes such as lactic acidosis or hyperchloremia, which may impact patient prognosis.^[10,11]^ Recent studies have suggested that anion gap may be a useful predictor of mortality in critically ill patients, including those with acute coronary syndromes.^[12]^

However, the relationship between anion gap and mortality in patients with CTO admitted to the ICU has not been extensively studied. The medical information mart for intensive care, version IV (MIMIC-IV) database, a large, publicly available dataset of ICU patients, provides an opportunity to investigate this relationship in a well-defined cohort.^[13]^ This study aims to evaluate the association between anion gap and 28-day all-cause mortality in patients with CTO admitted to the ICU, using data from the MIMIC-IV database. By identifying the predictive value of anion gap, this study may contribute to a better understanding of the factors influencing outcomes in this high-risk patient population and potentially guide clinical decision-making.

The primary objective of this study is to determine whether anion gap is independently associated with 28-day all-cause mortality in patients with CTO admitted to the ICU. Secondary objectives include assessing the impact of anion gap across different patient subgroups and exploring potential interactions with other clinical variables. The findings of this study could have implications for risk stratification and management strategies in patients with CTO in the ICU setting.

## 2. Methods

### 2.1. Study design and data source

This retrospective analysis leveraged data from version 3.1 of the MIMIC-IV database, comprising anonymized health records from ICUs at a specific medical center, covering the period from 2008 through 2019.^[13]^ The research adhered to ethical guidelines and privacy laws. The principal investigator, recognized for their completion of a relevant research training program and certified for database access (certification number 65828445), managed the data retrieval process. Given the use of publicly accessible and anonymized data, the study was exempt from institutional review board approval.

### 2.2. Inclusion and exclusion criteria

**Figure 1.**
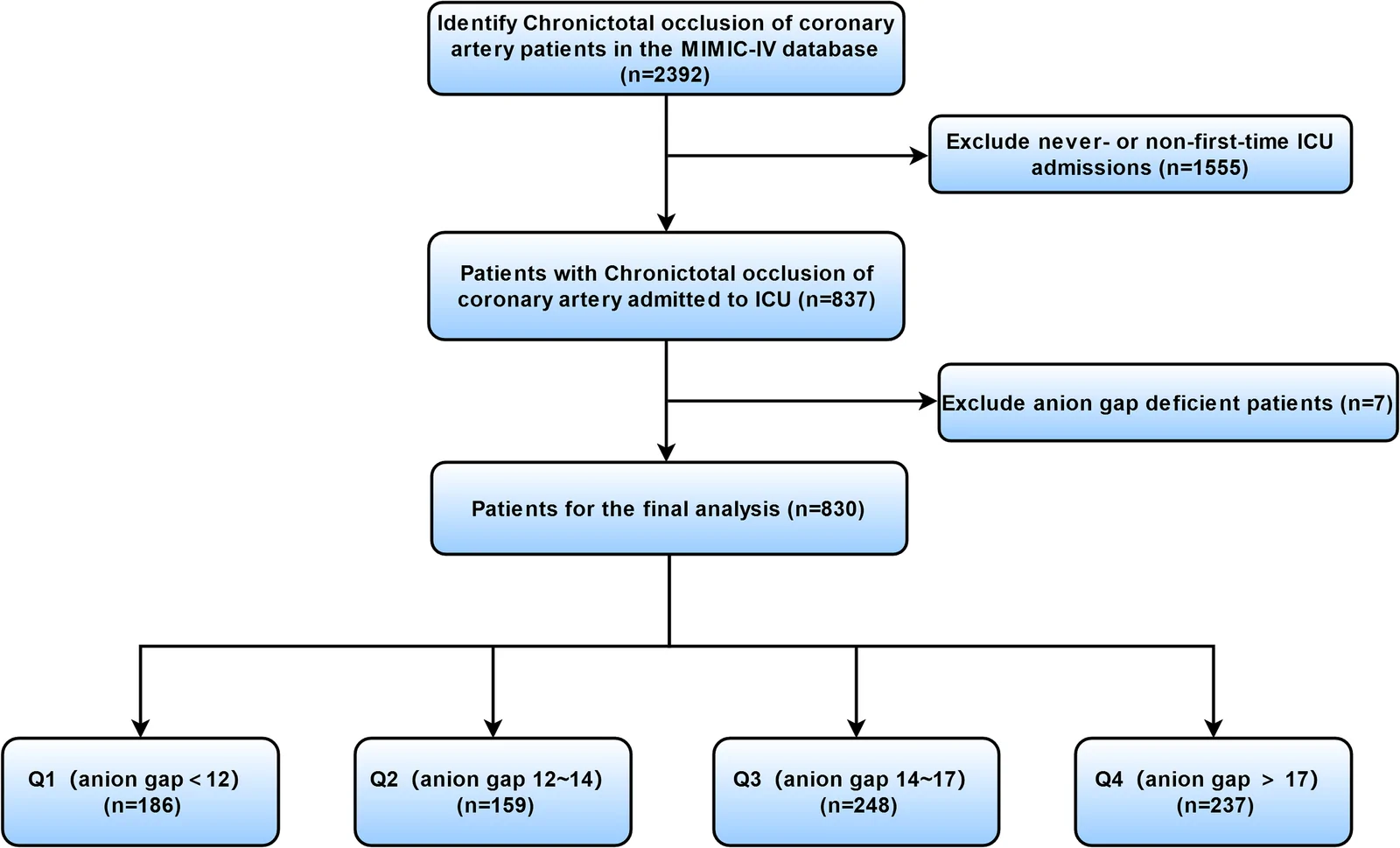
Flowchart of patient inclusion. ICU = intensive care unit, MIMIC-IV = medical information mart for intensive care, version IV, n = number of patients.

Patients were eligible for inclusion if they were diagnosed with CTO, as identified by International Classification of Diseases codes 4142 and I2582, and were admitted to the ICU for the first time. Additionally, patients had to be at least 18 years of age or older at the time of admission to ensure a homogeneous study population suitable for the analysis of the specified outcomes. Exclusions were applied to patients who did not meet the age requirement (< 18 years old), those who were admitted to the ICU more than once (n = 1555), or those with missing anion gap data within 24 hours of ICU admission (n = 7). This exclusion process aimed to reduce potential biases and confounding factors that could affect the validity of the study results. (Fig. 1)

### 2.3. Data collection

Data were extracted from the MIMIC-IV database within 24 hours of ICU admission. The variables of interest included demographic information (age, gender, race), clinical characteristics (body mass index [BMI], heart rate, systolic and diastolic blood pressure, temperature, oxygen saturation), and laboratory results (anion gap, bicarbonate, blood urea nitrogen, calcium, chloride, creatinine, sodium, potassium, international normalized ratio, prothrombin time, activated partial thromboplastin time, lactate, acute physiology and chronic health evaluation III [APSIII] score, simplified acute physiology score II [SAPS II], organ failure assessment score [OASIS], and sequential organ failure assessment [SOFA] score). Comorbidities such as heart failure, cerebrovascular disease, chronic pulmonary disease, and diabetes mellitus were also recorded.

### 2.4. Handling of missing data

Missing data were addressed using multiple imputation by chained equations, a statistical technique that creates multiple imputed datasets to account for the uncertainty due to missing values. This method is robust and suitable for handling missing data in observational studies, allowing for more accurate and reliable statistical analyses.

### 2.5. Statistical analysis

We performed statistical analyses using R Statistical Software (Version 4.2.2) and the Free Statistics Analysis Platform (Version 2.3; Beijing Ruisi Bai Data Technology Co., Ltd.).^[14]^ To evaluate the association between anion gap and 28-day all-cause mortality, we initially conducted univariate Cox regression analysis for each variable of interest. Subsequently, we constructed multivariate Cox regression models to assess the independent effect of anion gap on mortality while controlling for potential confounders. We developed 3 hierarchical models: Model 1 included adjustments for age, gender, race, and BMI; Model 2 further adjusted for clinical scores such as APSIII, SAPS II, OASIS, and SOFA; and Model 3 included additional adjustments for comorbidities like heart failure, cerebrovascular disease, chronic pulmonary disease, and diabetes mellitus. We verified the proportional hazards assumption for each model to ensure the validity of the Cox regression analysis.

To further explore the relationship between anion gap and mortality, we categorized anion gap into quartiles (Q1 < 12, Q2 12–14, Q3 14–17, Q4 > 17) and performed trend tests to assess the linearity of the association. Subgroup analyses were conducted to investigate potential interactions with age, gender, and diabetes. We considered a *P* value of < .05 to indicate statistical significance. The handling of missing data was addressed using multiple imputation by chained equations, which allowed us to create several imputed datasets and provide estimates that account for the uncertainty due to missing values. This method is recognized for its robustness in dealing with missing data in observational studies and was deemed suitable for our analysis.

## 3. Results

### 3.1. Patient characteristics

The study cohort included 830 patients with CTO admitted to the ICU. The baseline characteristics are summarized in Table 1. The mean age was 69.4 years, and 76.5% of the patients were male. The majority were nonwhite (72.0%), and the mean BMI was 29.4 kg/m^2^. Anion gap was categorized into quartiles: Q1 (< 12 mmol/L), Q2 (12–14 mmol/L), Q3 (14–17 mmol/L), and Q4 (> 17 mmol/L), with 186, 159, 248, and 237 patients in each group, respectively.

**Table 1:** Baseline characteristics.

Variables	Total (n = 830)	Q1 (n = 186)	Q2 (n = 159)	Q3 (n = 248)	Q4 (n = 237)	*P* value
Age (yrs), mean ± SD	69.4 ± 11.4	69.0 ± 10.2	69.2 ± 10.4	68.4 ± 11.2	71.0 ± 12.9	.082
Gender, n (%)						.273
Female	195 (23.5)	43 (23.1)	35 (22)	51 (20.6)	66 (27.8)	
Male	635 (76.5)	143 (76.9)	124 (78)	197 (79.4)	171 (72.2)	
Race, n (%)						.039
White	232 (28.0)	38 (20.4)	48 (30.2)	81 (32.7)	65 (27.4)	
Non-White	598 (72.0)	148 (79.6)	111 (69.8)	167 (67.3)	172 (72.6)	
BMI (kg/m^2^), mean ± SD	29.4 ± 6.2	29.8 ± 5.6	30.4 ± 6.3	29.7 ± 6.7	28.0 ± 5.8	< .001
Heart rate (beats/min), mean ± SD	80.5 ± 12.8	80.8 ± 9.8	78.5 ± 10.5	80.1 ± 12.8	81.9 ± 15.8	.062
SBP (mm Hg), mean ± SD	113.1 ± 12.6	112.6 ± 9.6	112.3 ± 11.3	114.4 ± 12.2	112.8 ± 15.6	.309
DBP (mm Hg), mean ± SD	60.5 ± 9.6	58.2 ± 7.5	58.7 ± 8.3	61.7 ± 9.9	62.2 ± 11.1	< .001
Temperature (°C), mean ± SD	36.7 ± 0.6	36.7 ± 0.4	36.7 ± 0.4	36.8 ± 0.4	36.5 ± 0.8	< .001
SpO_2_ (%), mean ± SD	97.2 ± 2.0	97.7 ± 1.4	97.5 ± 1.5	97.2 ± 1.8	96.6 ± 2.5	< .001
Glucose (mg/dL), mean ± SD	146.0 ± 45.8	132.2 ± 23.8	132.5 ± 31.0	144.1 ± 43.4	167.8 ± 59.7	< .001
Hematocrit, mean ± SD	30.7 ± 6.5	27.7 ± 5.2	29.6 ± 5.8	31.7 ± 6.4	32.7 ± 7.0	< .001
Haemoglobin (g/dL), mean ± SD	10.3 ± 2.3	9.3 ± 1.8	10.0 ± 2.1	10.7 ± 2.2	10.9 ± 2.5	< .001
Platelets (×10^9^/L), mean ± SD	171.6 ± 69.0	141.7 ± 55.3	160.2 ± 59.0	173.6 ± 64.1	200.5 ± 77.9	< .001
WBC (×10^9^/L), mean ± SD	14.5 ± 5.8	14.5 ± 5.2	14.1 ± 5.4	14.2 ± 5.6	15.1 ± 6.7	.268
Bicarbonate, mean ± SD	21.7 ± 3.7	23.1 ± 2.8	22.9 ± 2.8	22.1 ± 2.9	19.5 ± 4.5	< .001
BUN, median (IQR)	19.0 (15.0–28.0)	15.5 (13.0–20.0)	18.0 (14.0–22.0)	19.0 (15.0–27.0)	29.0 (19.0–44.0)	< .001
Calcium, mean ± SD	8.3 ± 0.7	8.3 ± 0.6	8.3 ± 0.6	8.4 ± 0.6	8.4 ± 0.8	.203
Chloride, mean ± SD	102.7 ± 5.0	105.5 ± 3.7	104.3 ± 4.1	102.8 ± 3.8	99.3 ± 5.6	< .001
Creatinine, median (IQR)	1.1 (0.8–1.4)	0.9 (0.8–1.1)	1.0 (0.8–1.2)	1.1 (0.9–1.4)	1.4 (1.0–2.3)	< .001
Sodium, mean ± SD	136.5 ± 3.7	136.8 ± 2.9	136.9 ± 3.2	136.8 ± 3.0	135.6 ± 4.9	< .001
Potassium, mean ± SD	4.7 ± 0.6	4.6 ± 0.4	4.5 ± 0.5	4.6 ± 0.7	4.9 ± 0.8	< .001
INR, median (IQR)	1.3 (1.2–1.5)	1.4 (1.3–1.5)	1.3 (1.2–1.5)	1.3 (1.2–1.5)	1.3 (1.1–1.5)	.001
PT, mean ± SD	16.5 ± 10.3	16.2 ± 7.9	15.9 ± 8.9	15.8 ± 5.9	18.0 ± 15.1	.074
APTT, median (IQR)	35.7 (30.0–65.2)	34.7 (29.2–40.5)	33.1 (28.9–41.7)	35.8 (30.4–64.6)	53.7 (32.0–105.4)	< .001
Lactate, mean ± SD	2.7 ± 1.8	2.5 ± 0.9	2.4 ± 1.0	2.6 ± 1.2	3.4 ± 2.8	< .001
APSIII, mean ± SD	40.7 ± 19.8	34.7 ± 14.9	36.3 ± 16.0	39.2 ± 18.1	49.9 ± 23.5	< .001
SAPS II score, mean ± SD	35.7 ± 11.9	35.0 ± 9.9	34.1 ± 10.0	34.2 ± 11.4	38.8 ± 14.2	< .001
OASIS, mean ± SD	30.8 ± 8.4	31.1 ± 7.4	30.0 ± 7.9	29.8 ± 8.0	32.3 ± 9.5	.004
SOFA score, mean ± SD	4.6 ± 2.9	4.7 ± 2.3	4.5 ± 2.8	4.3 ± 2.9	4.9 ± 3.4	.177
Heart failure						< .001
No	472 (56.9)	134 (72)	98 (61.6)	148 (59.7)	92 (38.8)	
Yes	358 (43.1)	52 (28)	61 (38.4)	100 (40.3)	145 (61.2)	
Cerebrovascular disease						.021
No	732 (88.2)	153 (82.3)	146 (91.8)	218 (87.9)	215 (90.7)	
Yes	98 (11.8)	33 (17.7)	13 (8.2)	30 (12.1)	22 (9.3)	
Chronic pulmonary disease						.434
No	640 (77.1)	145 (78)	124 (78)	197 (79.4)	174 (73.4)	
Yes	190 (22.9)	41 (22)	35 (22)	51 (20.6)	63 (26.6)	
Diabetes						.382
No	566 (68.2)	128 (68.8)	117 (73.6)	164 (66.1)	157 (66.2)	
Yes	264 (31.8)	58 (31.2)	42 (26.4)	84 (33.9)	80 (33.8)	

APSIII = acute physiology and chronic health evaluation III, APTT = activated partial thromboplastin time, BMI = body mass index, BUN = blood urea nitrogen, DBP = diastolic blood pressure, INR = International Normalized Ratio, IQR = interquartile range, n = number of patients, OASIS = organ failure assessment score, PT = prothrombin time, SAPS II = simplified acute physiology score II, SBP = systolic blood pressure, SD = standard deviation, SOFA = sequential organ failure assessment, SpO2 = oxygen saturation, WBC = white blood cell.

### 3.2. Univariate analysis

Univariate Cox regression analysis identified several variables significantly associated with 28-day all-cause mortality (Table 2). Specifically, for each unit increase in anion gap, the hazard ratio (HR) for 28-day mortality was 1.09 (95% confidence interval [CI] 1.05–1.13, *P* < .001). Other significant predictors included age, heart rate, systolic blood pressure, temperature, oxygen saturation, hematocrit, bicarbonate, blood urea nitrogen, calcium, chloride, sodium, international normalized ratio, prothrombin time, activated partial thromboplastin time, lactate, APSIII score, SAPS II score, OASIS score, SOFA score, and the presence of heart failure and cerebrovascular disease.

**Table 2:** A univariate Cox regression model evaluated the association between anion gap and 28-day all-cause mortality in patients with chronic total occlusion of the coronary artery.

Item	HR (95% CI)	*P* value
Age (per 1 yr)	1.06 (1.04–1.09)	< .001
Sex (male vs female)	0.63 (0.36–1.11)	.112
Race (non-White vs White)	0.82 (0.47–1.43)	.478
BMI (per 1 kg/m^2^)	0.93 (0.88–0.98)	.003
Heart rate (per 1 beat/min)	1.04 (1.02–1.05)	< .001
SBP (per 1 mm Hg)	0.94 (0.92–0.97)	< .001
DBP (per 1 mm Hg)	0.99 (0.97–1.02)	.698
Temperature (per 1 °C)	0.43 (0.34–0.53)	< .001
SpO_2_ (per 1%)	0.73 (0.67–0.79)	< .001
Hematocrit (per 1%)	1.01 (1.01–1.01)	< .001
Hemoglobin (per 1 g/dL)	1.02 (0.98–1.06)	.423
Platelets (per 10^9^/L)	0.97 (0.86–1.09)	.599
WBC (per 10^9^/L)	1.00 (1.00–1.01)	.047
Anion gap (per 1 unit)	1.09 (1.05–1.13)	< .001
Bicarbonate (per 1 mmol/L)	1.20 (1.16–1.24)	< .001
BUN (per 1 mg/dL)	0.77 (0.74–0.81)	< .001
Calcium (per 1 mg/dL)	1.03 (1.02–1.04)	< .001
Chloride (per 1 mmol/L)	0.36 (0.25–0.52)	< .001
Creatinine (per 1 mg/dL)	0.94 (0.90–0.98)	.006
Sodium (per 1 mmol/L)	1.33 (1.22–1.45)	< .001
Potassium (per 1 mmol/L)	0.96 (0.90–1.01)	.138
INR (per 1 unit)	1.84 (1.45–2.35)	< .001
PT (per 1 s)	1.21 (1.10–1.34)	< .001
APTT (per 1 s)	1.02 (1.01–1.03)	< .001
Lactate (per 1 mmol/L)	1.02 (1.02–1.03)	< .001
APSIII (per 1 point)	1.59 (1.48–1.71)	< .001
SAPS II score (per 1 point)	1.05 (1.04–1.06)	< .001
OASIS (per 1 point)	1.08 (1.07–1.10)	< .001
SOFA score (per 1 point)	1.13 (1.10–1.16)	< .001
Heart failure (yes vs no)	1.37 (1.27–1.49)	< .001
Cerebrovascular disease (yes vs no)	1.92 (1.13–3.27)	.016
Chronic pulmonary disease (yes vs no)	1.88 (0.97–3.63)	.061
Diabetes mellitus (yes vs no)	1.48 (0.84–2.61)	.179
Age (per 1 yr)	1.02 (0.58–1.79)	.947

APSIII = acute physiology and chronic health evaluation III, APTT = activated partial thromboplastin time, BMI = body mass index, BUN = blood urea nitrogen, CI = confidence interval, DBP = diastolic blood pressure, HR = hazard ratio, INR = International Normalized Ratio, OASIS = organ failure assessment score, PT = prothrombin time, SAPS II = simplified acute physiology score II, SBP = systolic blood pressure, SOFA = sequential organ failure assessment, SpO_2_ = oxygen saturation, WBC = white blood cell.

### 3.3. Multivariate analysis

Multivariate Cox regression analysis (Model 3) confirmed anion gap as a significant predictor of 28-day all-cause mortality (HR 1.15, 95% CI 1.10–1.20, *P* < .001). The risk increased across quartiles, with the highest risk observed in Q4 (HR 21.22, 95% CI 2.78–161.93, *P* = .003) compared to Q1 (Table 3).

**Table 3:** A multivariate Cox regression model evaluated the association between anion gap and 28-day all-cause mortality in patients with chronic total occlusion of the coronary artery.

Variable	No	Crude model		Model 1		Model 2		Model 3	
		HR (95% CI)	*P* value	HR (95% CI)	*P* value	HR (95% CI)	*P* value	HR (95% CI)	*P* value
Anion gap	830	1.20 (1.16–1.24)	< .001	1.23 (1.18–1.27)	< .001	1.15 (1.10–1.20)	< .001	1.15 (1.10–1.20)	< .001
Anion gap (quartile)									
Q1 (< 12)	186	1 (Ref)		1 (Ref)		1 (Ref)		1 (Ref)	
Q2 (12–14)	159	4.72 (0.53–42.20)	.165	4.45 (0.50–39.88)	.182	4.70 (0.52–42.12)	.167	5.72 (0.63–52.23)	.122
Q3 (14–17)	248	5.30 (0.65–43.07)	.119	4.95 (0.61–40.34)	.135	4.48 (0.55–36.51)	.162	5.20 (0.63–42.85)	.126
Q4 (> 17)	237	38.29 (5.27–277.91)	< .001	31.56 (4.32–230.46)	.001	16.58 (2.23–123.15)	.006	21.22 (2.78–161.93)	.003
Trend.test	830	3.89 (2.51–6.04)	< .001	3.54 (2.28–5.50)	< .001	2.42 (1.57–3.74)	< .001	2.57 (1.65–4.01)	< .001

Crude model (unadjusted).

Model 1: adjusted for age, gender, race, and BMI.

Model 2: Model 1 + APSIII score, SAPS II score, OASIS score, and SOFA score.

Model 3: Model 2 + heart failure, cerebrovascular disease, chronic pulmonary disease, and diabetes mellitus.

APSIII = acute physiology and chronic health evaluation III, BMI = body mass index, CI = confidence interval, HR = hazard ratio, OASIS = organ failure assessment score, SAPS II = simplified acute physiology score II, SOFA = sequential organ failure assessment.

### 3.4. Restricted cubic spline curve

**Figure 2.**
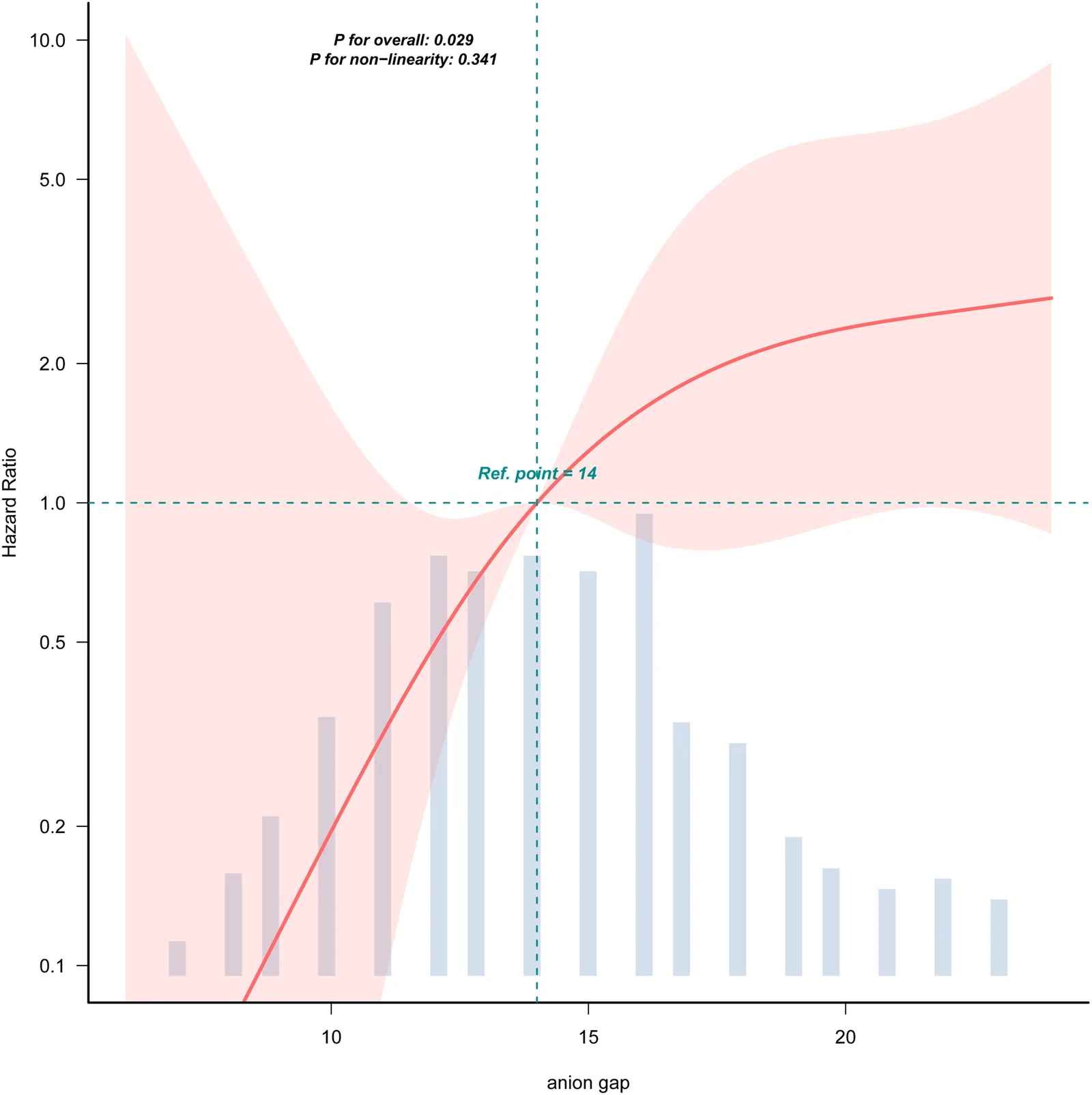
RCS curve for the anion gap (quartile): Q1 (< 12), Q2 (12–14) , Q3 (14–17) , Q4 (> 17). RCS = restricted cubic spline.

The restricted cubic spline curve for the anion gap (Fig. 2) demonstrated a nonlinear relationship with the HR, with a reference point at 14. The *P* value for overall association was .029, and for nonlinearity was .341, suggesting a significant but nonlinear relationship between anion gap and mortality.

### 3.5. Kaplan–Meier survival analysis

**Figure 3.**
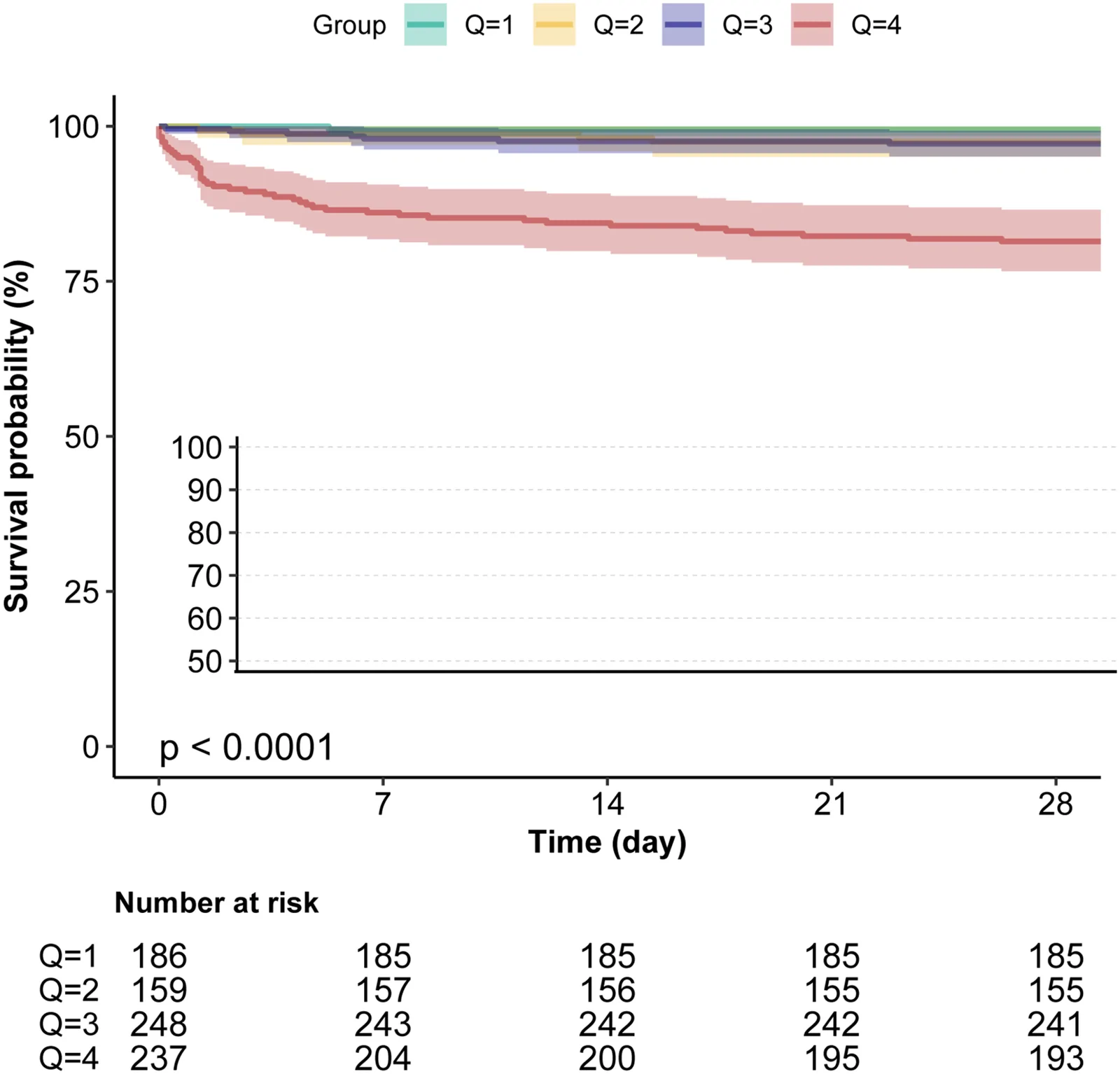
Kaplan–Meier survival analysis curves for 28-day all-cause mortality.

Kaplan–Meier survival analysis (Fig. 3) showed significant differences in survival probabilities across anion gap quartiles (*P* < .0001). Patients in Q4 had the lowest survival probability, highlighting the strong association between higher anion gap and increased mortality risk.

### 3.6. Subgroup and forest plot analysis

**Figure 4.**
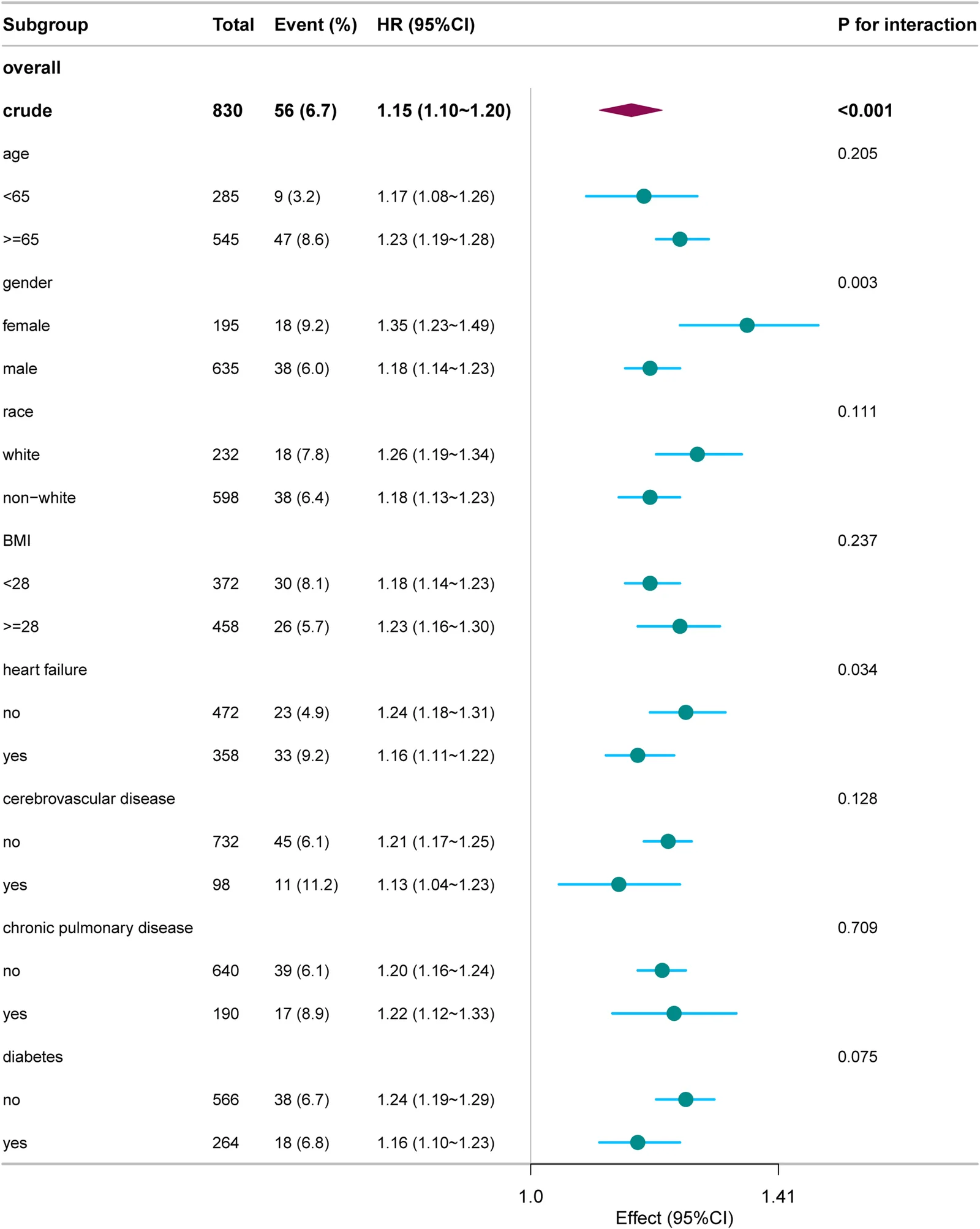
Forest plot for the subgroup analysis of the relationship between 28-day all-cause mortality and anion gap. BMI = body mass index, CI = confidence interval, HR = hazard ratio.

Subgroup analysis, as depicted in the forest plot (Fig. 4), revealed significant interactions between anion gap and age (*P* = .003), gender (*P* = .003), and heart failure (*P* = .034). The forest plot illustrates the HRs for 28-day all-cause mortality across different subgroups, with a clear trend of increased risk associated with higher anion gap levels. Notably, female patients and those with heart failure exhibited a higher risk associated with higher anion gap levels. The overall effect size across subgroups was consistent, indicating a robust association between anion gap and mortality.

## 4. Discussion

Our study reveals a significant association between anion gap and 28-day all-cause mortality in patients with CTO admitted to the ICU. The elevated anion gap, particularly in the highest quartile, is a robust predictor of increased mortality risk, even after adjusting for multiple confounders. This finding is clinically relevant and suggests that anion gap could serve as a useful biomarker for risk stratification in CTO patients.

The mechanism underlying the association between anion gap and mortality in CTO patients may involve several interrelated pathways.^[15]^ An elevated anion gap typically signifies a state of metabolic acidosis, which can arise from various causes, including lactic acidosis due to tissue hypoxia or sepsis.^[16]^ In the context of CTO, anion gap elevation might reflect the severity of myocardial ischemia and the subsequent release of acidic byproducts from ischemic myocardial tissue. This metabolic derangement could exacerbate cardiac dysfunction and contribute to a worse clinical outcome.^[17,18]^ Furthermore, an elevated anion gap could also indicate impaired renal function, which is common in critically ill patients and can lead to the accumulation of anions such as lactate and urea.^[19,20]^ Renal dysfunction is associated with worse outcomes in ICU patients and may be particularly detrimental in those with CTO due to the additional burden of cardiovascular instability.^[21]^ Another possible mechanism is the role of anion gap in reflecting the systemic inflammatory response.^[22]^ Inflammatory cytokines can increase anaerobic metabolism and lead to lactic acidosis, thereby raising the anion gap.^[23]^ This systemic inflammation can contribute to myocardial dysfunction and worsen cardiovascular prognosis in CTO patients.^[24,25]^

Our findings align with previous studies that have identified anion gap as a prognostic factor in critically ill patients. However, our study specifically focuses on CTO patients, providing novel insights into the role of anion gap in this high-risk group. The consistent association across different subgroups and the robustness of the relationship after multivariate adjustment strengthen the evidence for anion gap as a significant predictor of mortality in CTO patients.

The identification of anion gap as a significant predictor of mortality has important clinical implications. Monitoring anion gap could help clinicians in risk stratification and decision-making processes, potentially leading to more targeted interventions and improved patient outcomes. For instance, patients with elevated anion gap might benefit from closer monitoring, aggressive management of ischemia, and early intervention for complications such as sepsis or renal dysfunction.

## 5. Limitations

Several limitations should be considered when interpreting our results. Firstly, as a retrospective cohort study, our findings are subject to selection bias and confounding factors that could not be fully accounted for, even with extensive adjustments in our multivariate models. Secondly, the generalizability of our findings may be limited due to the specific population studied and the use of data from a single-center ICU database. Thirdly, the study did not include all potential confounding factors that could influence the relationship between anion gap and mortality, such as specific treatments received or genetic factors. Lastly, the nonlinear relationship between anion gap and mortality suggests that the impact of anion gap on mortality may vary across different levels, which could have implications for clinical decision-making.

Future research should aim to validate our findings in independent cohorts and explore the potential mechanisms underlying the association between anion gap and mortality in CTO patients. Additionally, prospective studies are needed to determine whether interventions targeting the anion gap can improve outcomes in this patient population. Further investigation into the nonlinear relationship between anion gap and mortality could also provide valuable insights into the optimal management strategies for patients with different levels of anion gap.

## 6. Conclusion

Our study demonstrates a significant association between anion gap and 28-day all-cause mortality in patients with CTO admitted to the ICU. These findings highlight the potential clinical utility of the anion gap as a prognostic marker in this high-risk patient population and warrant further investigation into its role in guiding clinical care. Understanding the mechanisms through which the anion gap influences mortality could lead to the development of novel therapeutic strategies and improve outcomes in CTO patients.

## Author contributions

**Conceptualization:** Jun Yang, Guang Tu.

**Data curation:** Jun Yang, Min Huang, Guofeng Zhu, Zhonglan Cai, Guang Tu.

**Formal analysis:** Min Huang, Guofeng Zhu, Zhonglan Cai.

**Funding acquisition:** Guang Tu.

**Investigation:** Jun Yang, Min Huang, Guofeng Zhu, Zhonglan Cai, Guang Tu.

**Methodology:** Jun Yang, Min Huang, Guofeng Zhu, Zhonglan Cai.

**Project administration:** Guang Tu.

**Supervision:** Jun Yang, Guang Tu.

**Writing – original draft:** Jun Yang, Min Huang, Guofeng Zhu, Zhonglan Cai, Guang Tu.

**Writing – review & editing:** Jun Yang, Guang Tu.
